# Tenosynovitis caused by *Mycobacterium marseillense*, initially identified as *Mycobacterium avium* complex using AccuProbe and COBAS TaqMan

**DOI:** 10.1186/s12879-021-06770-9

**Published:** 2021-10-23

**Authors:** Yusuke Nomura, Koh Okamoto, Yuki Ohama, Yoshimi Higurashi, Sohei Harada, Kyoji Moriya

**Affiliations:** 1 Department of Infection Control and Prevention, The University of Tokyo Hospital, Tokyo, Japan; 2 Department of Infectious Diseases, The University of Tokyo Hospital, 7-3-1 Hongo, Bunkyo-ku, 113-8655 Tokyo, Japan

**Keywords:** *Myobacterium marseillenese*, *Mycobacterium avium* complex, Non-tuberculosis mycobacteria (NTM), Tenosynovitis, MALDI-TOF MS, AccuProbe

## Abstract

**Background:**

*Mycobacterium marseillense* is a new species of the *Mycobacterium avium* complex. There has been only a few human infections caused by *M. marseillense* worldwide.

**Case presentation:**

We report a case of tenosynovitis caused by *M. marseillense* in an immunocompetent adult in Japan. The isolate was initially identified as *M. intracellulare* using commercial real time polymerase chain reaction assays and later identified as *M. marseillense* with sequencing of the the *rpoB* and *hsp65* regions, and matrix-assisted laser desorption ionization–time of flight mass spectrometry (MALDI-TOF MS).

**Conclusions:**

This is the first case reporting on *M. marseillense* generating a positive result with commercial real time PCR assays targeting MAC. Human infections associated by *M. marseillense* might be underreported due to similarities with *Mycobacterium intracellulare*. To accurately identify *M. marseillese*, MALDI-TOF MS might provide a rapid and reliable method.

## Background

The *Mycobacterium avium* complex (MAC) is the most common non-tuberculosis mycobacteria (NTM) causing human infections; these include chronic pulmonary infections in adults and lymphadenitis in children. *M. avium* and *M. intracellulare* are the best-known representative species of the MAC. However, with advance in molecular analyses, multiple species have been recognized as members of the MAC, resulting in frequent emendation in the taxonomy of this species complex [1]. Although there is no widely accepted definition with regard to what constitutes the MAC, a recent review proposed a definition of the MAC based on phylogenetic analyses that includes 12 valid published species [2]. *Mycobacterium marseillense* is among these [1]; however, little is known about its microbiological and clinical features. We here report a case of tenosynovitis with *M. marseillense* that was initially identified as *M. intracellulare* using commercial real time polymerase chain reaction (PCR) assays.

## Case presentation

In March 2018, an 85-year-old man with no medical history was admitted after a 6-month history of worsening pain and swelling in the right wrist. Magnetic resonance imaging showed a large region of tenosynovitis from the distal forearm to the palm, and a biopsy of the synovial membrane was performed. Histological testing showed granulomatous inflammation. After nine weeks of culture on Ogawa medium (Kyokuto Pharmaceutical Industrial CO., Japan), smooth non-pigmented colonies were observed. The colony was positive for Ziehl Neelsen stain, suggesting acid fast bacilli. Initial testing with AccuProbe (Hologic, Marlborough, MA) was positive for MAC, with a value of 197,546 relative light units (RLU). COBAS TaqMan MTB/MAI (Roche Diagnostics, Switzerland), another real-time PCR test, was positive for *M. intracellulare* as well. However, further identification of the isolate using matrix-assisted laser desorption ionization–time of flight mass spectrometry (MALDI-TOF MS; MALDI Biotyper Version 2.0, Bruker Daltonics, US) suggested that the isolate was *M. marseillense*, with a score of 2.023 indicating a probable species identification. Because of this discordance, the *rpoB* and *hsp65* regions were sequenced [3, 4]. In the Basic Logical Alignment Search Tool (BLAST) analysis, both sequences showed a 99.7% (E value, 0.0) and 100.0% (E value, 0.0) alignment respectively with those of *Mycobacterium marseillense* (GenBank accession number CP023147.1).

Drug susceptibility testing was performed by a broth microdilution test, BrothMIC NTM (Kyokuto Pharmaceutical Industrial Co., Japan). Based on the breakpoints proposed by the Clinical and Laboratory Standards Institute [5], the isolate was sensitive to clarithromycin and amikacin. Subsequently, clarithromycin, ethambutol and rifampicin were initiated, and symptoms subsided gradually over the following month. Rifampicin was discontinued due to neutropenia after 2 months. Unfortunately, the patient died from an unrelated cause six months after the initiation of treatment.

## Discussion

Here we describe a case of tenosynovitis of the wrist by *M. marseillense* in an immunocompetent adult. Thus far, five human infections of *M. marseillense* have been reported from Italy, China and the US (Tables 1, 2) [6–10].

**Table 1:** Human Infections with *Mycobacterium marseillesnse*

No.	Age	Sex	Country	Immunocompromising condition	Site of infection	Identification methods	Antibiotics therapy	Duration of therapy	Outcome	References
1	56	M	China	Systemic lupus erythematosus	Pneumonia	Gene Sequencing (16 S rRNA, ITS,*hsp65*)	None^a^	Not documented	unknown	[6]
2	65	M	Italy	None	Pneumonia	Gene Sequencing (*rpoB*, ITS), Genotype CM/AS (misidentified as MIN)	1. RFP, INH, AMK 2. LVFX, TRD, AZM 3. EB, RFP, AZM	54 months^b^	Cured	[7]
3	4	F	Italy	None	Lymphadenitis	Gene Sequencing (ITS), Genotype CM	CAM, RFP + EB^c^	6 months	Cured	[8]
4	59	F	China	None	Skin infection of face	Gene Sequencing (16 S rRNA, *hsp65*, *rpoB*)	1. RFP, INH, EB, PZA 2. CAM, RFP, EB 3. CAM, MFLX, AMK	15 months^d^	Cured	[9]
5	73	M	US	Renal transplant due to polycystic kidney disease	Tenosynovitis of wrist	Not documented	AZM, EB, Rifabutin	6 months	Cured	[10]
6	85	M	Japan	None	Tenosynovitis of wrist	MALDI TOF-MS, Gene Sequencing (*rpoB*, *hsp65*), Accuprobe (positive for MAC)	1. CAM, EB, RFP 2. CAM, EB	5 months^e^	Died from unrelated cause	This Study

M, male; F, female; RFP, rifampin; INH, isoniazid; AMK, amikacin; LVFX, levofloxacin; TRD, terizidone; AZM, azithromycin; EB, ethambutol; CAM, clarithromycin; MFLX, moxifloxacin

^a^Macrolide and rifampin was planned but canceled due to an acute intracranial hemorrhage

^b^Regimen 1 for 12 months, regimen 2 for 36 months, regimen 3 for 6 months

^c^Ethanbutol was later added due to delay of the surgical scar

^d^Regimen 1 for 10 months, regimen 2 for 3 months, regimen 3 for 2 months

^e^ Regimen 1 for 2 months, regimen 2 for 3 months

**Table 2:** Drug susceptibility and minimum inhibitory concentration (µg/mL) of *M. marseillense* isolates

No.	SM	EB	KM	RFP	RBT	LVFX	MFLX	CPFX	CAM	TH	AMK	LZD	ST	INH	AZM	Reference
1	–	R >32	–	R 16	–	–	R 8	R 16	S 1	–	S 32	R 32	R 32/608	–	–	[6]
2	S	R	–	S	–	–	–	–	–	–	–	–	–	S	–	[7]
3	–	–	–	–	–	–	–	–	–	–	–	–	–	–	–	[8]
4	–	R	–	R	–	–	I	–	S	–	S	–	–	–	S	[9]
5	–	–	–	–	–	–	S	–	S	–	S	S	–	–	–	[10]
6	1	1	1	≦0.03	0.03	0.5	–	–	S 0.06	4	S 1	–	–	–	–	This Study

SM, streptomycin; EB, ethambutol; KM, kanamycin; RFP, Rifampin; RBT, rifabutin; LVFX, levofloxacin; MFLX, moxifloxacin; CPFX, ciprofloxacin; CAM, clarithromycin; TH, ethionamide; AMK, amikacin; LZD, linezolid; ST, trimethoprim/sulfamethoxazole; INH, isoniazid; AZM, azithromycin. S, sensitive; I, intermediate; R, resistant. The drug susceptibility for case No.1- 5 are described as stated in the literatures [6–10]

Accurate diagnosis of the MAC to the species level is challenging as they are very similar in biochemical features [2]. In addition, they are closely related genetically, with 98–99% similarity in the 16S rRNA gene [2]. Thus, a combination of ribosomal and/or housekeeping genes has been used for identification of novel strains and species. In the reported cases, the identification was mostly made by sequencing multiple regions, such as 16S rRNA, internal transcribed spacer-1 region (ITS), *hsp65*, and *rpoB*. In our case, sequencing of the *rpoB* and *hsp65* region and MALDI-TOF MS successfully identified the strain as *M. marseillense*. However, sequencing is not readily available in clinical settings; this poses a challenge to clinicians.

Commercial probes for the clinical important mycobacteria are widely used in clinical settings. Among these, AccuProbe is an FDA-approved polymerase chain reaction (PCR) test used to identify several clinically important mycobacteria, including *M. tuberculosis* complex, *M. avium, M. intracellulare*, and *M. kansasii* [11]. The identification is based on the hybridization of specific DNA probes to the target 16 S rRNA. By using chemiluminescence, the DNA-rRNA hybrid molecule is detected and measured in relative light units (RLU). A reliable species identification for NTM requires a cutoff value over 30,000 RLU and may require even higher values, above 80,000 for MAC [11]. Nonetheless, cross reactions with other mycobacteria including *M. arosiense*, *M. chimaera*, *M. nebraskense*, *M. saskatchewanense*, and *M. colombiense* have been reported [11, 12]. To our knowledge, this is the first report of the positive reaction for MAC with AccuProbe and COBAS TaqMan by *M. marseillense* in the literature. Of note, one study reported the misidentification of *M. marseillense* as *M. intracellulare*, using a different commercial identification kit (Genotype Mycobacterium CM/AS assay, Hain Lifescience GmbH, Germany), leading to a delay in accurate diagnosis [7].

MALDI-TOF MS may be of great help for mycobacterial identification in clinical microbiology laboratories. In a study of 125 isolates from 27 different NTM species, identification using MALDI-TOF MS with a cut-off score of 1.7 had a 94.4 % (118/125) agreement between 16 S rRNA and *hsp65* sequencing to the species level [13]. The seven isolates that could not be identified using MALDI-TOF MS were *M. massiliense*, (n = 4; not included in the database) and *M. gordonae* (n = 3; with no peak or reliable result) [13]. *M. massilense* was not included at the time of the study but has later been added in the most recent database (MALDI Biotyper Version 6). Other studies have also reported similar results reporting a 92.0 to 97.6 % agreement between MALDI-TOF MS and either 16S rRNA, *rpoB* or *hsp65* sequencing [14, 15].

In conclusion, we report on a case of tenosynovitis of the wrist by *M. marseillense* in an immunocompetent man. Given the limited availability of sequencing in clinical settings, cases of *M. marseillense* infection might have been misidentified and, therefore, underreported. To understand the epidemiology of *M. marseillense* and its role in human infections, accurate identification is crucial, and MALDI-TOF MS might provide a rapid and reliable identification when sequencing specific regions is not readily available.

## Data Availability

The datasets used and/or analysed during the current study available from the corresponding author on reasonable request.

## Abbreviations

MAC: Mycobacterium avium complex
NTM: Non-tuberculosis mycobacteria
PCR: Polymerase chain reaction
MALDI-TOF MS: Matrix-assisted laser desorption ionization–time of flight mass spectrometry
BLAST: Basic logical alignment search tool
RLU: Relative light units
